# The Possible Clinical Predictors of Fatigue in Parkinson's Disease: A Study of 135 Patients as Part of International Nonmotor Scale Validation Project

**DOI:** 10.4061/2011/125271

**Published:** 2011-12-05

**Authors:** Vinod Metta, Kartik Logishetty, P. Martinez-Martin, Heather M. Gage, P. E. S. Schartau, T. K. Kaluarachchi, Anne Martin, Per Odin, P. Barone, Fabrizio Stocchi, A. Antonini, K. Ray Chaudhuri

**Affiliations:** ^1^National Parkinson Foundation Centre of Excellence, King's College Hospital, King's College London, Denmark Hill Campus, 9th Floor, Ruskin Wing, > London SE5 9RS, UK; ^2^University Hospital Lewisham, London SE13 6LH, UK; ^3^University of Surrey, Surrey GU2 7XH, UK; ^4^Alzheimer Disease Research Unit, CIEN Foundation, Carlos III Institute of Health, Alzheimer Center Reina Sofia Foundation, Madrid, Spain; ^5^Department of Neurology, University Hospital, Lund University, Lund, Sweden; ^6^Department of Neurology, Central Hospital, Reinkenheide, Bremerhaven, Germany; ^7^Department of Neurological Sciences, University of Naples Federico II, Via S. Pansini 5, 80131 Naples, Italy; ^8^Department of Neurology, IRCCS San Raffaele, 00163 Rome, Italy; ^9^Department of Neurology, IRCCS San Camillo Venice and University of Padua, 35122 Padua, Italy

## Abstract

Fatigue is a common yet poorly understood and underresearched nonmotor symptom in Parkinson's disease. Although fatigue is recognized to significantly affect health-related quality of life, it remains underrecognised and empirically treated. In this paper, the prevalence of fatigue as measured by a validated visual analogue scale and the Parkinson's disease nonmotor symptoms scale (PDNMSS) was correlated with other motor and nonmotor comorbidities. In a cohort of patients from a range of disease stages, occurrence of fatigue correlated closely with more advanced Parkinson's disease, as well as with depression, anxiety, and sleep disorders, hinting at a common underlying basis.

## 1. Introduction

Parkinson's disease (PD) is a progressive neurodegenerative disorder which is known to be associated with nonmotor symptoms (NMS) including dribbling of saliva, constipation, depression, sleep disorders, apathy, hallucinations, and dementia [1]. NMS of PD contributes significantly to health-related quality of life (HrQoL) and some NMS such as constipation, hyposmia, rapid eye movement behavior disorder (RBD), fatigue, and depression may be markers of a preclinical stage of PD [2, 3]. In spite of its clinical and patient-related importance, NMS remain undeclared and often undertreated in primary or secondary care [4].

Fatigue is now recognized as a key NMS in PD. It may be present at diagnosis and can complicate late disease and become an overwhelming problem for patients and relatives [5–7]. Indeed, fatigue impacts most dimensions of HrQoL even as a factor independent of depression and disability [8–10]. The largest holistic study of NMS in PD, the PRIAMO study, reported fatigue to be prevalent in 58.1% of a population sample of 1072 patients ranging from 37.7% in early (Hoehn and Yahr (HY) stage 1) PD to 81.6% in advanced (HY stage 5) [11]. Other studies have suggested a prevalence between 33% to 58%, although the method of diagnosis and definition of “fatigue” is heterogeneous [7, 12–16]. However, there is a poor correlation between the severity of mental and physical fatigue suggesting independent aetiologies [17]. Since there is as yet no biomarker of fatigue, patient-reported questionnaires remain the mainstay of measuring and diagnosing fatigue. Specific scales for clinical assessment of fatigue have now been validated for PD and can be used in the clinic while “holistic” nonmotor scales can be used to explore the relationship of other key NMS of PD such as depression, apathy, excessive daytime sleepiness, and fatigue. The Parkinson's nonmotor group (PDNMG) led pivotal studies validating the first nonmotor questionnaire (NMSQuest) and subsequently, the nonmotor scale (NMSS) for PD [18–20]. Fatigue is a key component of domain 2 of the NMSS. It is scored based on the multiplication of its severity and its frequency—a measure that was validated based on the collection of data from an unselected cohort of PD patients from a number of international centers. 

Fatigue in PD occurs independent of motor severity. There is a paucity of research exploring the treatment of fatigue in PD. There is conflicting evidence regarding the efficacy of levodopa in treating fatigue [21, 22] while it may be alleviated using methylphenidate [23]. Independent of PD, large cohort [24–26] studies have associated fatigue with a more sedentary lifestyle. Only one study, which evaluated patients with PD, found that lower levels of physical activity, poorer physical function, and less frequent strenuous exercise were associated with increased fatigue (see the work of Garber and Friedman [27]). A study on rats which had 6-OHDA lesioning [28] has suggested a link between increased exercise and reduced loss of striatal dopamine concentrations, with another study showing that later intervention with exercise did not improve deficits [29]. However, no animal studies to date have demonstrated an improvement of fatigue with exercise, and there is no evidence yet to recommend the efficacy of exercise therapy on fatigue in PD in humans. 

In this study, we have analysed the prevalence of fatigue from the composite data set used to validate the nonmotor symptom scale. We attempted to explore if other measures used in the study contributed or could be marked as “predictors” of fatigue in this population. We also aimed to discern whether disease severity or pharmacological treatment of PD affected the prevalence of fatigue. An improved understanding of this poorly researched area of nonmotor symptoms in PD could then guide a holistic treatment strategy.

## 2. Methods

### 2.1. Design

This data is obtained from the database related to the validation study of the NMSS [18] which was an international, cross-sectional, open, multicentre, one point-in-time evaluation with retest study. Details of the administration of the scales have been published previously.

### 2.2. Patients

Consecutive patients with PD (patients with Parkinsonism were excluded), of all age groups and disease severity, satisfying the UK PD Brain Bank criteria for diagnosis of idiopathic PD were included after attending relevant outpatient clinics [30]. Patients with a diagnosis of parkinsonism due to alternative causes were excluded.

Patients were recruited from specialist movement disorder clinics at the National Parkinson Foundation centre of excellence at King's College and Lewisham Hospitals (UK) and also local Care of the Elderly clinics. This ensured that we recruited a range of patients from the composite group. Cases with disease severity spanning Hoehn and Yahr (HY) stages 1 to 5 were studied, and we also attempted to include a proportion of untreated cases. Datasets were included where fatigue data was available and computable. 

Only patients able to provide informed consent were selected for the study and demented patients were excluded. The latter would mean that the clinician would exclude patients with significant cognitive impairment that

- affected their ability to provide informed consent,
- affected their ability to complete self-reported items.

The data presented relates to 135 patients included largely from the King's/Lewisham, German and Italian sites.

## 3. Ethical Approval

Central ethical approval for the full study was initially obtained via the research ethics committee at University Hospital Lewisham and subsequently all centres obtained site specific ethical approval.

## 4. Procedure

The scales (listed below) and the nonmotor symptom questionnaire used in the study were applied following a standardized protocol, always in the same order, and there was no reported patient fatigue while completing the scales [3, 18]. Patient and carers took approximately 25 minutes to complete the questionnaire while the investigator-led instruments took 40 minutes to complete.

## 5. Assessments

The researcher then completed the following battery of standard assessment measures:

- standard demography form,
- unified Parkinson's disease rating scale (UPDRS) [31],
- cumulative illness rating scale-geriatrics [32] (CIRS-G to measure physical comorbidity),
- Hoehn and Yarh scale [33],
- frontal assessment battery [34] (FAB),
- nonmotor symptom scale [18].

Sections 3 and 4 of the UPDRS were applied. These domains evaluate motor examination and complications of therapy, respectively. The frontal assessment battery is a short scale exploring assessing cognitive and behavioural function, as a measure of executive capacity in PD. 

In addition, the patient (assisted by the research nurse if necessary) completed the following self-assessments:

- PDQ-8 [35] (a specific instrument for assessment of health-related quality of life in PD),
- a fatigue-visual analogue scale (VAS-F) [36],
- hospital anxiety and depression Scale (HADS) [37] (a self-administered instrument developed for the detection of mood disorders in nonpsychiatric outpatients attending hospital consulting rooms).

The total time required for a single assessment was approximately 65 minutes per patient. 

### 5.1. Fatigue

For this study the VAS was used as a measure of convergent validity with the fatigue section of the NMSS. Fatigue was assessed using the fatigue VAS validated specifically for PD. Patients were asked about the severity of perceived fatigue by means of visual analogue scale (VAS) where 0 represented the worst imaginable fatigue state to 100 representing no fatigue at all [37].

### 5.2. The NMS Scale

The NMS scale is composed of 30 items grouped in 9 domains: cardiovascular, sleep/fatigue, mood/apathy, perceptual problems, attention/memory, gastrointestinal, urinary, sexual function, and miscellaneous. Each item is scored for frequency (ranging from 1 (rarely) to 4 (very frequent)) and intensity (ranging from 0 (none) to 3 (severe)). The product of these scores gives the item severity score (0 to 12). Domains and NMSS total scores are obtained by the sum of items scores. The scale has been extensively validated in two large international studies exceeding 700 patients [3, 18].

### 5.3. Inclusion/Exclusion Criteria

All patients with established diagnosis of PD satisfying the UK Brain bank criteria were included while all cases of Parkinsonism due to other causes were excluded [13]. Additionally, after data had been collected, patients whose clinical assessments indicated dementia, severe depression (HADS > 13), or major sleep disturbances were excluded as these factors may confound assessment of fatigue.

### 5.4. Statistics

Descriptive statistics were used for the study variables as needed. For comparisons, the Pearson's chi-squared, Mann-Whitney test (to test between two groups), and Kruskal-Wallis test (to compare more than two groups) were applied, as the variables did not meet the assumptions for use of parametric statistics. Looking for a balance between error types I and II adjustment for multiple comparisons, the Benjamini-Hochberg method was applied. Association of nonlinear relationships was analyzed by Spearman rank correlation coefficient (*r*_*s*_) with an alpha level set at 0.0001 but with a Bonferroni downward adjustment for multiple comparisons. The statistical software SPSS 19.0 (IBM, USA) was used for data analysis.

## 6. Results

A total of 135 patients met eligibility criteria with complete datasets. Demographic details and summary of measures are shown in Tables 1–5. Patients had a mean age of 69.7 ± 10.52 years and an age range of 35 to 88 yrs (24.4% TD, 28.9% AD, 46.6% mixed). However, as this was a clinic-based study, only 2% were HY stage 5 while the majority were between HY stages 2-3 (Table 4). The breakdown of NMSS scores in each domain is shown in Table 6. 

Data from the application of the fatigue-specific visual analogue scale showed no significant differences between males (1) and females (2) using the Mann-Whitney test (male 62 ± 20.4 versus female 63.1 ± 19.2). Analysis of fatigue scores (grouped as mild, moderate, or severe) using HY staging of disease progression (HY 1–2.5 = 7 (mild fatigue); HY 3 = 8 (moderate fatigue); HY 4-5 = 9 (severe fatigue)) showed a significant correlation (*P* = 0.004) using the Kruskal-Wallis test as seen in Table 3. No significant differences were observed in fatigue scores between the different drug treated and drug naïve patients using the Mann-Whitney test. Correlation measures using Spearman rank correlation coefficient were used between the various variables and fatigue scores and are listed in Table 7. 

Correlation measures using the Spearman rank correlation coefficient associating measured variables from UPDRS, CIRS, HADS, FAB, PDQ-8, Hoehn & Yahr, NMSQuest, and NMSS instruments are also shown in Table 7. Increased fatigue was independently correlated strongly (*r* < −0.30, *P* < 0.0001) with HADS anxiety and depression domains, FAB score, total NMSQuest score, total NMSS score, HRQo measured by PDQ-8, and NMS sleep and mood domains.

## 7. Discussion

Fatigue was noted by James Parkinson (1817) in his original description of the disorder, but it is only in 1993 that studies began describing its prevalence, progression, and impact and characterising fatigue [7].

Studies since then have suggested that sleep disorders, medications, and depression may be possible secondary causes of fatigue in individuals with PD [6]. The prevalence of autonomic impairment, nocturnal sleep disturbances, and depression have been found to exacerbate the subjective perception of fatigue. However, fatigue occurs in patients with PD independent of sleep dysfunction and in nondepressed patients, suggesting that these factors may not be the only contributors to the high prevalence of fatigue in PD patients. Indeed, population-based studies in PD report that sleep disorders and in particular excessive daytime somnolence (EDS) do not account for fatigue in the majority of PD subjects. This relationship is, however, clouded by evidence showing that certain medications used to treat PD are associated with EDS [6]. 

The PRIAMO study showed that fatigue is present in patients presenting at Hoehn and Yahr stage 1 of the disease while the percentage of patients with fatigue rises as the disease progresses to stage 5 [11]. It is likely that in the majority of PD patients, fatigue is intrinsic to the disease.

Our current study was aimed at exploring these various issues, in particular by using PD-specific fatigue measures which were used as secondary variable measure in the original validation study of NMSS, and employing correlation measures with seemingly confounding variables. The patient base was “real life” and representative of a PD population.

We found no difference in fatigue scores between subtypes of PD. Akinesia dominant cases had similar levels of fatigue compared to tremor dominant types, and fatigue levels were similar between male and female patients. However, using Kruskall-Wallis comparative measures, fatigue levels worsened significantly with worsening disease severity as measured by HY stage and graded as mild, moderate, and severe (Table 2). This observation is in line with the recently reported PRIAMO study where fatigue levels were reported incrementally as HY stages increased [11]. However, it is to be noted that a substantial proportion of mild PD cases, including drug naïve PD patients, experienced some level of fatigue. 

The level of fatigue between drug naïve cases and those treated with either mono or combination therapy of antiparkinsonian agents were similar and provides an indirect support for the observation that fatigue in PD appears to be unaffected by conventional PD therapy. However, we observed a trend (although nonsignificant) of better fatigue scores in those treated by combined levodopa and dopamine agonists (fatigue score of 71.3 ± 19.1 in untreated PD versus fatigue score of 60.6 ± 20.6 in combined therapy). At least one study has suggested that pergolide, an ergot dopamine agonist may improve fatigue in PD although controlled studies are lacking [38]. 

Finally, in an attempt to unravel the positive correlations of fatigue and other measures in PD, a detailed correlation analysis was undertaken (Table 7). The key findings were that anxiety and depression as measured by the HADS anxiety and depression subscales, and the mood domain of NMSS showed a robust association with fatigue scores on VAS. Sleep dysfunction was also associated highly significantly with fatigue while HY score also registered a significant association. All together this translated to a robust association with health-related quality of life as measured by PDQ-8. 

The association of fatigue with sleep dysfunction and anxiety and depression appears confirmatory with other observations as quoted previously while the lack of association with age, sex, and pattern of PD is also in line with established views on fatigue [6]. Statistically the dominant predictive factors of fatigue emerging from this study are depression, anxiety, and sleep dysfunction. This was our a priori hypothesis and may support a view held by Shulman et al. who suggested that nonmotor symptoms of PD such as fatigue, depression, and pain could share the same pathogenic origin [39]. 

Indeed, a recent study suggests that reduced serotonin (and perhaps dopamine) in certain areas of the brain may provide the pathophysiological basis of fatigue in PD [40]. Pavese et al. [40] used ^11^C-DASB imaging to demonstrate reduced serotonin transporter binding in the caudate, putamen, ventral striatum, and thalamus in PD patients with fatigue. Although there was no difference between nigrostriatal dopamine levels as measured by ^15^F-dopa values, reduced uptake in the insular cortex of PD patients with fatigue points to a link between fatigue and loss of extrastriatal dopaminergic function. Methylphenidate, a dopamine transporter blocker, has been shown to be effective in improving fatigue in PD patients [23]. While SSRIs have been used to treat chronic fatigue, and anecdotally to address fatigue in PD, the ^11^C-DASB findings suggest that treatment strategy should aim to restore serotonin levels rather than inhibit its transport (via the serotonin transporter, SERT). 

Although it is clinically difficult to separate fatigue and sleep given the difficulty a patient may have in distinguishing the two, several studies have suggested that fatigue is an independent nonmotor symptom unrelated to sleepiness [7, 16]. Sleepiness in PD has been associated to damage to central arousal systems by neuronal loss and Lewy bodies in several areas including serotonergic neurons in the raphe median nucleus. 

Isolating depression from fatigue in PD can also be challenging. Although it has been shown that fatigue occurs more in the depressed patient than in the general population [41, 42], this relationship is obscured by varying definitions of “fatigue” in the literature. More so, symptoms of fatigue and anergia contribute to the disability of depression. However, there are reports demonstrating that PD patients may experience depression and/or sleepiness without fatigue [5]. Depression has long been associated with widespread serotonergic loss [43, 44]. One small study has shown that nortriptyline, a tricyclic antidepressant with moderate antiserotonin receptor effects, improved symptoms of fatigue [45]. 

Although it is unlikely that one neuronal hormone, be it serotonin or dopamine, is responsible for fatigue, sleepiness, anxiety, or depression in PD, this study shows a commonality between these nonmotor symptoms, and is thus consistent with the hypothesis proposed by Shulman et al. Less robust association was observed for instance with cardiovascular NMS such as orthostatic tolerance, sexual function, pain, hyperhidrosis, attention, and memory functions (Table 7). Further studies must be designed to disentangle the specific relationship of these issues with fatigue, including the role of sleep pattern in fatigued PD patients, and explore alternative strategies to target the role of low central serotonin levels in these patients. 

In conclusion, this study suggests that fatigue is an important independent nonmotor symptom of PD and is associated with depression, anxiety, and sleep disorders. Fatigue appears to be widespread irrespective of the motor stage of PD and has a close correlation with quality of life in people with Parkinson's. 

## Figures and Tables

**Table 1:** Distribution of disease severity as measured by Hoehn and Yahr stage (H & Y stage).

H & Y stage	Patients (*n*)	Percent (%)
1	16	11.85
1.5	17	12.59
2	22	16.30
2.5	20	14.81
3	42	31.11
4	15	11.11
5	3	2.22

**Table 2:** Distribution of patients after H & Y stage grouped by severity.

H & Y categories	Patients (*n*)	Percent (%)
Mild	75	55.56
Moderate	42	31.11
Severe	18	13.33

Subdivisions: H & Y 1–2.5 = (mild); 3 = (moderate); 4 + 5 = (severe).

**Table 3:** Analysis of fatigue scores using Hoehn and Yahr staging of disease progression showed a significant correlation (*P* = 0.004) using the Kruskal-Wallis test.

H & Y categories	Mean fatigue score	Std. Dev.
Mild	67.52	18.33
Moderate	56.83	17.16
Severe	54.94	26.44

**Table 4:** The distribution of antiparkinsonian therapy used in the patients studied.

Therapies	Patients (*n*)	Percent (%)
Drug naïve	12	8.89
Levodopa monotherapy	50	37.04
DA monotherapy	11	8.15
Levodopa + DA	61	45.19
Others	1	0.74

DA: dopamine agonists.

**Table 5:** A unified table showing demographic values and assessment scores in this study.

Variable	Patients (*n*)	Mean	SD	Minimum	Maximum
Age	135	69.74	10.52	25	88
Duration	135	5.78	5.19	0	32
Agedx	135	63.88	11.34	29	65
UPDRS 3	130	16.13	8.01	2	44
UPDRS 4	132	2.92	3.03	0	13
Dysk & Flct	132	2.32	2.74	0	12
CIRS Total	135	4.74	2.84	0	15
VAS-F	135	62.52	19.90	10	100
HADS Anx	135	10.73	4.79	0	21
HADS Dep	135	10.19	4.62	1	21
FAB Total	134	14.79	2.83	3	18
PDQ-8	134	28.19	17.82	0	78.13

SD: standard deviation, duration = duration of disease, agedx: age at diagnosis. Dysk & Flct: dyskinesia and fluctuation, UPDRS 3 and 4: unified Parkinson's disease rating scale domains 3 & 4, CIRS: cumulative illness rating scale, VAS-F: fatigue-specific visual analogue scale, HADS Anx & Dep: hospital anxiety and depression rating scale anxiety and depression domains, FAB: frontal assessment battery.

**Table 6:** Table showing nonmotor scale (NMSS) data distribution including subitem scores.

Variable	Patients (*n*)	Mean	Std. Dev.	Min	Max
GI	135	4.38	5.14	0	23
Urinary	135	6.44	7.01	0	36
CV	133	2.60	4.04	0	21
Sexual function	135	3.08	5.66	0	24
Sleep/fatigue	135	11.11	9.03	0	48
Perception	135	1.81	4.43	0	36
Mood	135	10.56	14.34	0	72
Attention/memory	135	5.97	8.03	0	36
Misc.	135	6.66	7.38	0	36
NMSS total	133	52.96	41.52	0	243

GI: gastrointestinal, CV: cardiovascular, Misc.: miscellaneous, NMSS total: total score on NMSS.

**Table 7:** Correlation measures using Spearman rank correlation coefficient measures between the various variables and fatigue scores.

Variable	*r*	*P*
Age	—	N.S.
**UPDRS domain 3**	**−0.2380**	=**0.0064**
UPDRS domain 4	−0.1968	0.0237
CIRS total	—	N.S.
CIRS domain 1	—	N.S.
**HADS anxiety domain**	**−0.3948**	<**0.0001**
**HADS depression domain**	**−0.4171**	<**0.0001**
**Frontal assessment battery**	**0.3374**	=**0.0001**
**NMSQuest total**	**−0.3122**	=**0.0003**
NMSQuest sleep domain	−0.2823	=0.0009
Other NMSQuest domains	—	N.S.
Hoehn & Yahr staging	−0.2882	=0.0007
**PDQ-8**	**−0.367**	<**0.0001**
**NMSS total**	**−0.3924**	<**0.0001**
NMSS cardiovascular domain	−0.2985	=0.0004
NMSS sexual function domain	−0.2576	=0.003
**NMSS sleep domain**	**−0.3908**	<**0.0001**
**NMSS mood domain**	**−0.3766**	<**0.0001**
NMSS attention/memory domain	−0.2574	=0.003
NMSS misc. domain	−0.2666	0.002
Other NMSS domains	—	N.S.

The key correlations (*P* < 0.0001) are highlighted (N.S.: not significant, UPDRS: unified Parkinson's disease rating scale, CIRS: cognitive impairment rating scale, HADS: hospital anxiety and depression scale, NMSQuest: nonmotor symptom questionnaire, NMSS: nonmotor symptom scale).
